# A hybrid systems biology and systems pharmacology investigation of Zingerone’s effects on reconstructed human epidermal tissues

**DOI:** 10.1186/s43042-021-00204-6

**Published:** 2021-12-13

**Authors:** Elham Amjad, Babak Sokouti, Solmaz Asnaashari

**Affiliations:** Biotechnology Research Center, Tabriz University of Medical Sciences, Tabriz, Iran

**Keywords:** Zingerone, Skin-aging, Systems biology, Systems pharmacology, Interleukin, Gene–disease association, Gene–chemicals network, Similar compounds, Skin disorder

## Abstract

**Background:**

As individuals live longer, elderly populations can be expected to face issues. This pattern urges researchers to investigate the aging concept further to produce successful anti-aging agents. In the current study, the effects of Zingerone (a natural compound) on epidermal tissues were analyzed using a bioinformatics approach.

**Methods:**

For this purpose, we chose the GEO dataset GSE133338 to carry out the systems biology and systems pharmacology approaches, ranging from identifying the differentially expressed genes to analyzing the gene ontology, determining similar structures of Zingerone and their features (i.e., anti-oxidant, anti-inflammatory, and skin disorders), constructing the gene–chemicals network, analyzing gene–disease relationships, and validating significant genes through the evidence presented in the literature.

**Results:**

The post-processing of the microarray dataset identified thirteen essential genes among control and Zingerone-treated samples. The procedure revealed various structurally similar chemical and herbal compounds with possible skin-related effects. Additionally, we studied the relationships of differentially expressed genes with skin-related diseases and validated their direct connections with skin disorders the evidence available in the literature. Also, the analysis of the microarray profiling dataset revealed the critical role of interleukins as a part of the cytokines family on skin aging progress.

**Conclusions:**

Zingerone, and potentially any constituents of Zingerone (e.g., their similar compound scan functionality), can be used as therapeutic agents in managing skin disorders such as skin aging. However, the beneficial effects of Zingerone should be assessed in other models (i.e., human or animal) in future studies.

## Background

The skin, consisting of the epidermis, dermis, and subcutaneous layers, is the body’s largest organ and forms a physical barrier between the external environment and the internal environment that protects and maintains. As the population ages, the epidermis and dermis are the primary targets for various changes that encourage the development of novel anti-aging therapeutic agents [1]. Physical and chemical environmental factors such as UV radiation and xenobiotics play significant roles in the oxidative stress of skin cells by altering the involved signaling pathways, immunosuppression, and producing reactive oxygen species (ROS) and photosensitivity diseases [2]. Various skin anti-aging treatment procedures are available, including topical retinoids, peels (e.g., salicylic acid), botulinum neurotoxin, soft tissue fillers, collagens, hyaluronic acid, autologous fat, allogenic and synthetic products, lasers, surgical procedures, and endocrinological therapies, as well as other alternatives such as phytohormones [3]. The reports showed that the use of natural compounds has promising results in reducing UV-induced effects of skin aging, which have made them play the primary role in cosmetic-related sciences [4].

Zingerone (4-(4-hydroxy-3-methoxyphenyl)-2-butanone) is an inexpensive and nontoxic phenolic alkanone structure derived from Ginger (Zingiber officinale Rosc.), which is a widely used herb in pharmaceutical and food industries throughout the world (e.g., China, Greece, and India) [5–7]. Zingerone is a result of gingerol dehydration while being cooked or dried [5]. The main pharmacological properties of Zingerone include anti-oxidative, immune-stimulant, anti-inflammatory, and anti-cancer effects [8]. Previous studies reported the anti-ultraviolet B (UVB) radiation activity of Zingerone in protecting the epidermis [9].

Zingerone likely acts as a neuroprotective agent by blocking the apoptotic pathway, thus preventing oxidative stress and limiting inflammation [10]. It is thought that polyphenolic compounds called Zingerone are present in ginger and have potent anti-oxidant properties, exhibit free radical scavenging activity, and provide resistance to oxidative stress [11]. Various protective effects of Zingerone have been reported in lead-induced toxicity [12], streptozotocin/high fat diet-induced type 2 diabetes [13], rheumatoid arthritis [14], lipopolysaccharide-induced oxidative stress, DNA damage, cytokine storms [15], experimental colon carcinogenesis [16], alloxan-induced oxidative stress [17], cyclophosphamide‑induced organ toxicity [11], and cisplatin (cis-diamminedichloroplatinum (II))-induced jejunal toxicity [18]. Zingerone substantially decreased NF-κB, TGF-β, TNF-α, IL-1β, IL-6, and Hs-CRP levels while considerably increasing IL-10 levels and restoring anti-oxidant enzyme levels [14]. Thus, when given to animals (e.g., Wistar rats) exposed to the above-mentioned toxicities and diseases, Zingerone may reduce oxidative stress, inflammation, and multi-organ damage.

To the best of the authors’ knowledge, no investigation has used systems biology and systems pharmacology approaches to determine significantly differentially expressed genes between control tissues and those treated by Zingerone. In this study, we used the available microarray gene expression profiling datasets to meet this aim. The functional and cellular mechanisms of identified genes were also inspected. Then, various network analyses, including gene–disease and gene–chemicals, were performed. Finally, the effects of chemical and herbal compounds similar to Zingerone’s structure were studied in detail, and the validation of potential significant genes was reviewed based on evidence found in the literature.

## Methods

**Fig. 1:**
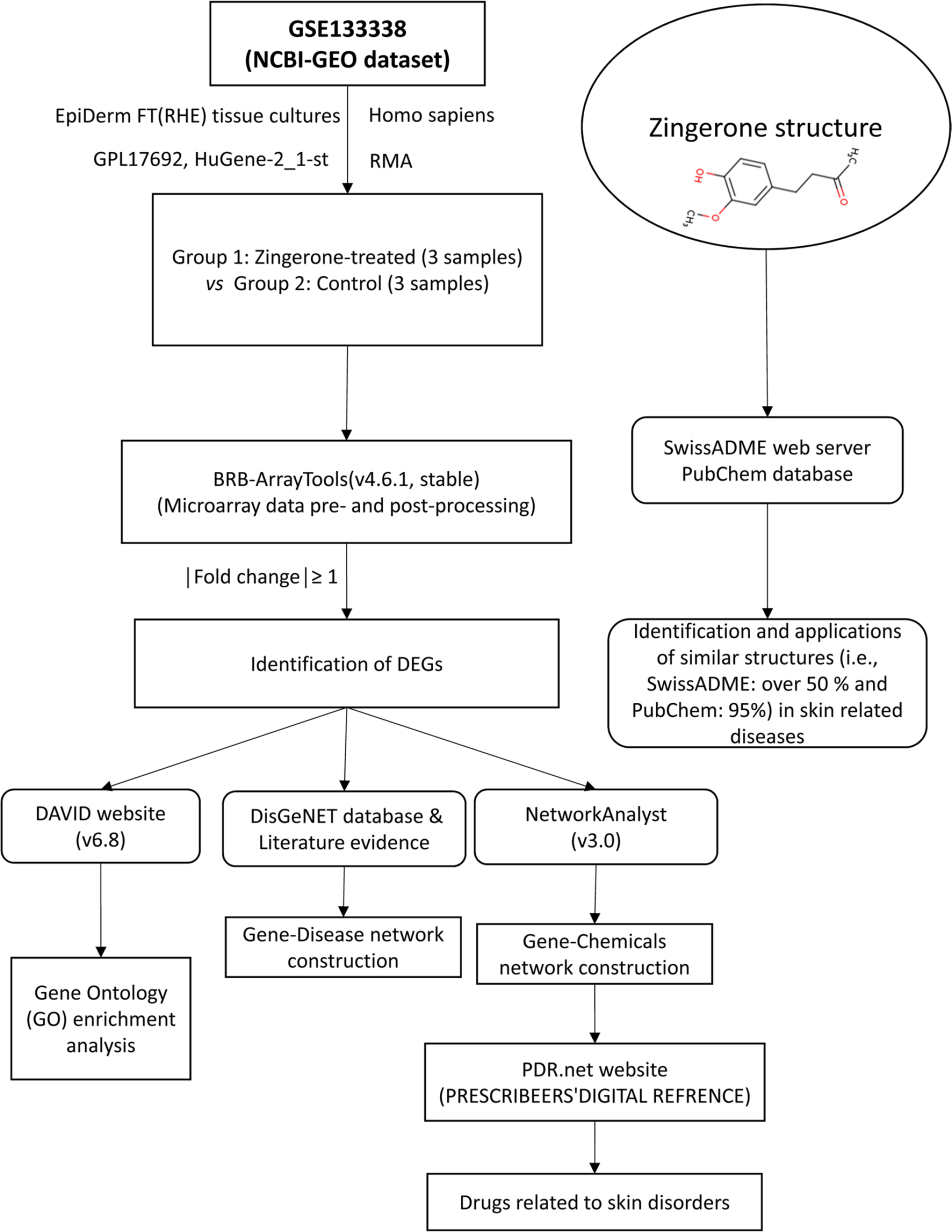
The overall workflow of the systems biology and systems pharmacology approaches

A summary of the current research workflow is illustrated as a flowchart diagram, as shown in Fig. 1.

### Data source

The dataset used in this study is publicly available from the gene expression omnibus (GEO) database repository of the national center for biotechnology information (NCBI) (i.e., https://www.ncbi.nlm.nih.gov/geo/) with the GPL17692 [HuGene-2_1-st] Affymetrix Human Gene 2.1 ST Array [transcript (gene) version] platform. The only available GEO dataset, GSE133338, included six reconstructed human epidermal (RHE) tissues treated by Zingerone (n = 3) and water control (n = 3), as shown in Fig. 1.

### Differentially expressed genes (DEGs) between two types of tissues

Dr. Richard Simon and the BRB-ArrayTools Development Team developed a genomics analytical tool, BRB-ArrayTools (v4.6.1, stable version), to determine potent DEGs. Several steps, including GEO dataset import, gene filtering (i.e., |fold change|≥ 1), and normalization (i.e., quantile normalization), and annotation (i.e., “pd.hugene.2.1.st” R package [19]), were required to identify the significant DEGs. The BRB-ArrayTools used the gcrma (guanine-cytosine robust multi-array analysis) algorithm to map the probe intensities into their corresponding gene expression values by discarding existing noise. The comparison between two treated groups resulted in the identification of significant DEGs using values of 10,000 and 1 for univariate permutation tests and the threshold of fold change. The obtained DEG results were significant at *p* ≤ 0.05.

### Gene ontology and functional enrichment analyses

DAVID v. 6.8 (Database for Annotation, Visualization, and Integrated Discovery), which is freely available at http://david.abcc.ncifcrf.gov/summary.jsp, provided the evaluation of the gene ontology (GO). This evaluation included cellular components, molecular functions, and biological processes of DEGs [20, 21]. The threshold for the EASE score of a modified Fisher exact *p* value was set to its default value of 0.1.

### Effectiveness and similarity structure analyses of Zingerone

**Fig. 2:**
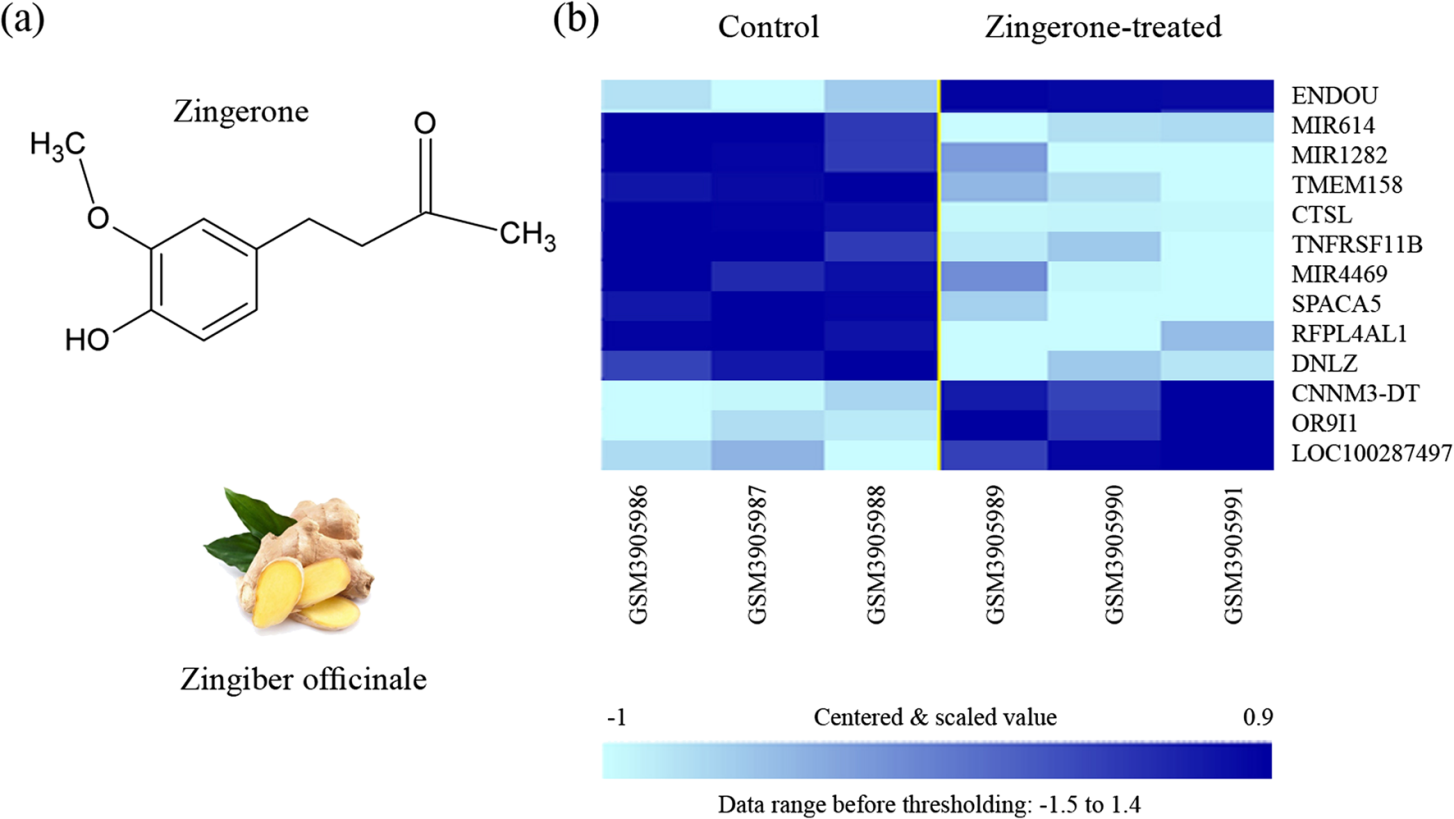
**a** Chemical structure of zingerone [4-(4-hydroxy-3-methoxyphenyl) butan-2-one] as a *zingiber* constituent. **b** DEGs obtained from BRB-ArrayTools analysis

The canonical SMILES (simplified molecular-input line-entry system) string for Zingerone structure (shown in Fig. 2a) was obtained from the PubChem compound database (https://pubchem.ncbi.nlm.nih.gov/compound) [22]. Each string of SMILES was used for PubChem similarity structure and ADME prediction using online web server tools, i.e., SwissADME [23–25], SwissSimilarity [26], and SwissTargetPrediction [27]. The SwissADME web server covers the drug design and discovery by computing several parameters, including physicochemical descriptors, ADME-related parameters, pharmacokinetic properties, drug-likeness effect, and studying medicinal chemistry friendliness of SMILES structure.

The similarity criteria for the PubChem similarity structure and the SwissSimilarity tools were 0.92 and 0.50, respectively. Finally, we screened the literature for any existing evidence for functionalities of similar compounds.

### Gene–disease and gene–chemicals network analyses

We extracted the related diseases with evidence according to the significant genes obtained through the user-friendly DisGeNET (i.e., http://www.disgenet.org/) platform, derived from the literature on human gene–disease associations [28]. Additionally, the involvement of obtained DEGs affected by Zingerone treatment in several specific diseases (and possibly in skin-related disorders) was thoroughly screened through the literature studies to confirm and validate whether such genes are expressed as potential biomarkers of the disease. Furthermore, we constructed and analyzed the gene–chemicals network from the NetworkAnalyst 3.0 webserver to reveal potent associations between genes and compounds available in literature [29].

## Results

Since missing any possible genes could affect the final results, all of the genes included in the microarray dataset are considered. The data preprocessing and class comparison approach using a two-sample t test revealed a total of sixty probe IDs, from which only thirteen DEGs were available as annotated genes. Four genes were downregulated, and nine were upregulated (as shown in Fig. 2b), along with their gene expression levels between control and Zingerone-treated samples.

The DAVID functional annotation tool revealed that two cellular components (i.e., (i) GO:0005615, and extracellular space with four involved genes TNFRSF11B, CTSL, ENDOU, and SPACA5 and (ii) GO:0005576, an extracellular region with three involved genes TNFRSF11B, CTSL, and ENDOU) were found significant. The kappa values of 1.00 and 0.80 indicated a very high level of similarity between the two GO terms. Moreover, the statistical measurement values for GO:0005615 and GO:0005576 were fold enrichments of 7.73 and 4.85, Bonferroni values of 0.09 and 0.72, Benjamini values of 0.088 and 0.60, and false discovery rate (FDR) values of 0.088 and 0.6, respectively. The detailed inspection of GO terms shows that their corresponding child terms have various relationships and cross-references with GO:0005576 and GO:0005615.

The relationships involving the extracellular space suggest that interleukin-35 complex, interleukin-27, interleukin-23, and interleukin-12 were part of GO:0005615. Moreover, the relationships involving the extracellular region were mainly part of the extracellular isoamylase complex, extracellular ferritin complex, extracellular space, extracellular vesicle, and extracellular matrix, to mention a few.

Four types of interleukins (i.e., interleukin-1, interleukin-6, interleukin-34, and interleukin-34 alpha) had direct relationships with cross-references of GO:0005615 (for more information, refer to https://www.ebi.ac.uk/QuickGO/GTerm?id=GO:0005615). Several interleukin types, including interleukin-12 alpha, interleukin-23 alpha, interleukin-17, interleukin-1 alpha, interleukin-1 beta, and interleukin-6, were related to cross-references of GO:0005576 (derived from https://www.ebi.ac.uk/QuickGO/GTerm?id=GO:0005576).

**Fig. 3:**
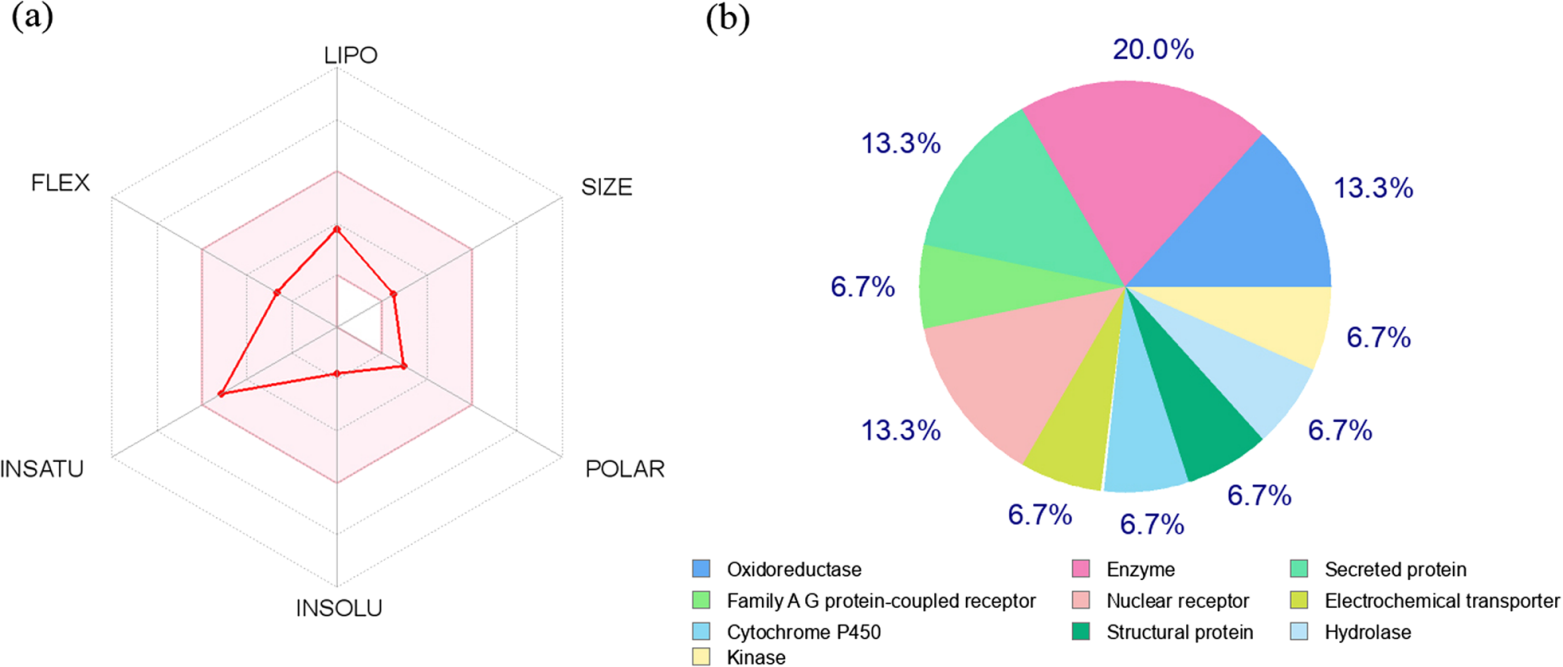
**a** The bioavailability radar chart of zingerone structure obtained from SwissADME prediction, **b** the small molecule protein targets for zingerone structure obtained from SwissTargetPrediction web server

The assessment of the absorption, distribution, metabolism, and excretion (ADME) of the Zingerone structure using the bioavailability radar (Fig. 3a) indicates the high bioactive drug-likeness property and represents the lipophilicity, molecular weight, solubility, and flexibility properties positioned in the pink area. Moreover, physicochemical and lipophilicity properties (Lipinski’s rule of five) (i.e., molecular weight: 194.23 g/mol, number of rotatable bonds: 4, number of H-bond acceptors: 3, number of H-bond donors: 1, and consensus Log Po/w: 1.79) show no violations. The water solubility parameters, including Log S (ESOL), Log S (Ali), and Log S (SILICOS-IT), demonstrate very soluble, very soluble, and soluble features, respectively. Also, the pharmacokinetics properties reveal a high level of gastrointestinal absorption and only CYP1A2 inhibitory function among other cytochrome enzymes inhibitors.

The SwissTargetPrediction analysis results in a total of 100 target proteins for Zingerone, among which enzymes, secreted proteins, nuclear receptors, and oxidoreductases make up the highest percentages (Fig. 3b). A further inspection of similar structures through the SwissSimilarity and PubChem uncovered two FDA-approved drugs (i.e., Nabumetone and Masoprocol), two experimental drugs (i.e., Matairesinol and 3-(4-hydroxyphenyl)-1-(2,4,6-trihydroxyphenyl)propane-1-one), and three Zingiber constituents (i.e., 6-Shogaol, 6-Paradol, and 6-Gingerol) (listed in Table 1). The inspection also revealed their validated anti-inflammatory and anti-oxidant properties, as well as the confirmed skin disorders, such as skin anti-aging, through the evidence provided in the literature.

**Table 1:** The list of identified compounds from SwissSimilarity and PubChem structure similarity accompanied by their anti-oxidant, anti-inflammatory, and related skin disorders

Item	Compound	Similarity	Status	Antioxidant	Anti-inflammatory	Skin disorders
1	Nabumetone	0.8^1^	FDA approved	[62]	[63]	Skin injury [64]
2	Masoprocol	0.672^1^	FDA approved	[65]	[65]	Sun damage (actinic keratosis) [66]
3	Matairesinol	0.815^2^	Experimental	[67]	[68]	Skin aging [69]
4	3-(4-hydroxyphenyl)-1-(2,4,6-trihydroxyphenyl)propan-1-one (Phloretin)	0.726^2^	Experimental	[70]	[70]	Skin damage [71, 72]
5	6-Shogaol	> 0.95^3^	Zingiber constituents	[73, 74]	[73, 74]	Skin aging [75]
6	6-Paradol	> 0.95^3^	Zingiber constituents	[76, 77]	[77]	Skin cancer [76]
7	6-Gingerol	> 0.92^3^	Zingiber constituents	[74]	[74]	Skin aging and damage [75, 78]

^1^The obtained results are from SwissSimilarity through performing ligand-based virtual screening of combined FDA approved drugs (n = 1516) of small molecules (> 50%)

^2^The obtained results are from SwissSimilarity through performing ligand-based virtual screening of combined experimental drugs (n = 4788) of small molecules (> 50%)

^3^The obtained results are from PubChem structure similarity through Tanimoto threshold of 95% and 92%

**Fig. 4:**
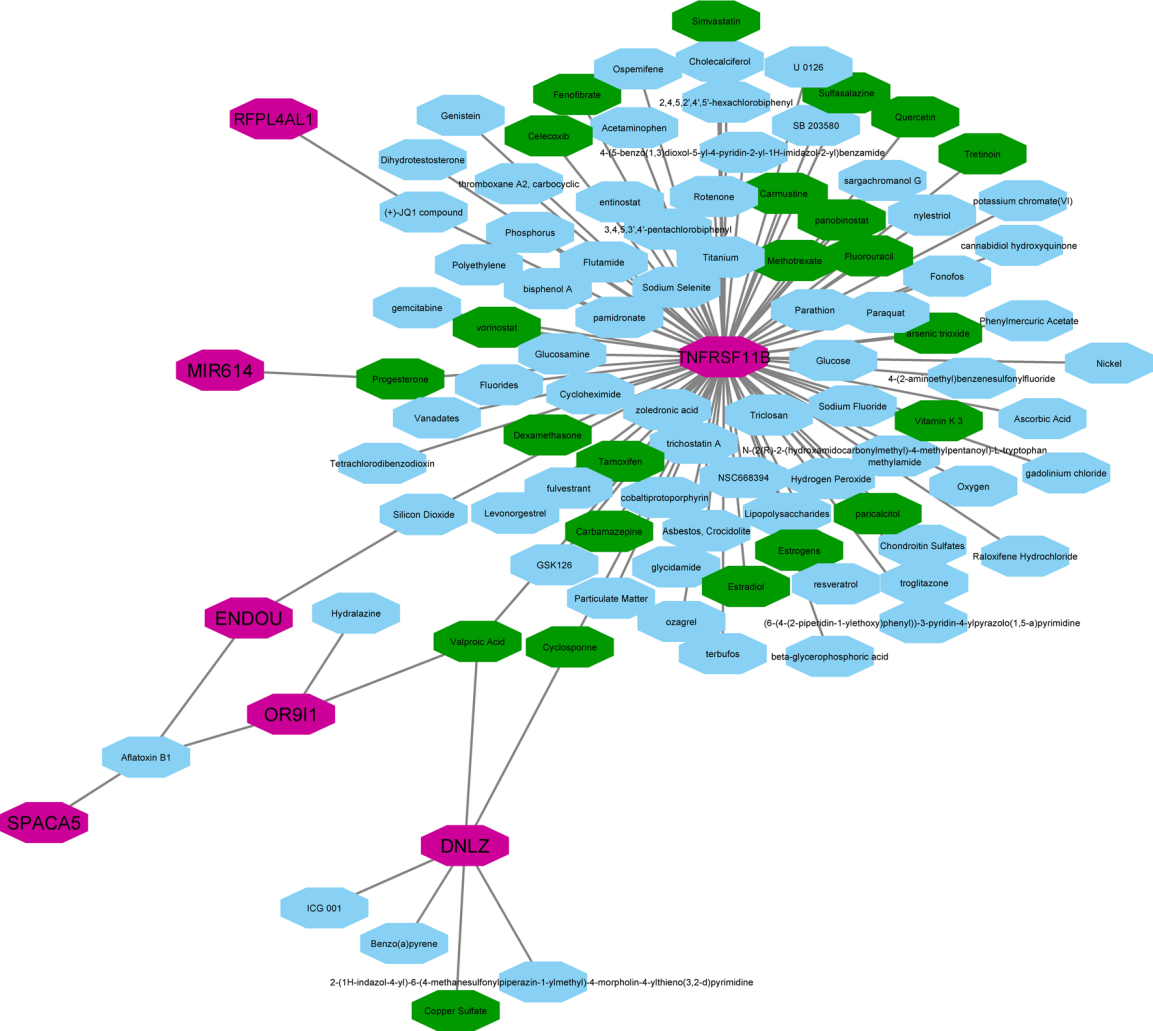
The gene–chemicals association network illustrating compounds directly connected with chemicals for skin-related disorders in green color. The involved genes are shown in purple

The gene–disease relationship outcomes from the DisGeNET (accompanied by the relevant evidence from the literature) are summarized in Table 2. The results indicate that the DEGs influence various diseases, including skin-related disorders. Furthermore, the construction of the gene–chemicals network shows the involvement of seven DEGs that are directly associated with different chemicals. Only four of them (i.e., TNFRSF11B, DNLZ, OR9I1, and MIR614) are related to twenty-three skin-related compounds (shown in Fig. 4).

**Table 2:** Validation of significant DEGs using DisGeNET and literature evidence for their related diseases. log_2_|FC| values are derived from ExAtlas meta-analysis web server

Gene Symbols	log_2_|FC|	DisGeNET	Literature
CTSL	1.47^1^	Disease: Meningioma Disease Class: Neoplasms; Nervous System Diseases [79]	Skin Proteome and Degradome [80]
		Disease: Liver carcinoma Disease Class: Digestive System Diseases; Neoplasms [81]	Keratinocytes and perturbation of hair Follicle cycling [82]
		Disease: Hereditary Diffuse Gastric Cancer Disease Class: Digestive System Diseases; Neoplasms [83]	Mouse skin carcinogenesis [84]
RFPL4AL1 *Paralog of FPL4A gene	1.56^1^	Disease: Malignant neoplasms Class: Neoplasms Disease: Colorectal carcinoma Class: Digestive System Diseases; Neoplasms Disease: Primary malignant neoplasm Class: Neoplasms [85]	Malignant melanoma [86] COVID-19 disease [87] Hepatocellular carcinoma [88]
TNFRSF11B	1.62^1^	Disease: Hyperphosphatasemia with bone disease Disease Class: Musculoskeletal Diseases [89]	Skin inflammation [90]
		Disease: Osteoporosis Disease Class: Nutritional and Metabolic Diseases; Musculoskeletal Diseases [91]	Skeletal dysplasias [92]
		Disease: Rheumatoid Arthritis Disease Class: Skin and Connective Tissue Diseases; Musculoskeletal Diseases; Immune System Diseases [93]	
SPACA5	1.64^1^	No matches found	Bladder cancer [94]
TMEM158	1.88^1^	Disease: Neoplasms [95]	Pediatric localized scleroderma skin [96]
		Disease: Carcinogenesis Disease Class: Pathological Conditions, Signs, and Symptoms; Neoplasms [97]	Anaplastic large cell lymphoma [97]
		Disease: Ovarian neoplasm Disease Class: Neoplasms; Female Urogenital Diseases and Pregnancy Complications; Endocrine System Diseases [98]	Skin [99]
DNLZ	1.93^1^	Disease: Neoplasms [100]	Immune evasion[101]
		Disease: Liver carcinoma Disease Class: Digestive System Diseases; Neoplasms [102]	Fibrogenic responses [103]
		Disease: Tumor cell invasion [104]	Psoriasis [105]
MIR614	2.00^1^	Disease: ovarian neoplasm; malignant neoplasm of ovary; carcinoma, ovarian epithelial Disease Class: Neoplasms; Female Urogenital Diseases and Pregnancy Complications; Endocrine System Diseases [106]	Psoriasis [107] Suppression of stromal interferon signaling [108]
MIR1282	2.00^1^	No matches found	Breast cancers [109] Hepatocellular carcinoma [110] Associated with immune organs and immunocytes [111]
MIR4469	2.58^1^	Disease: Malignant neoplasm of breast; breast carcinoma Disease Class: Neoplasms; Skin and Connective Tissue Diseases [112]	Atherosclerosis [113] Laryngeal carcinoma cells [114]
LOC100287497 (SEPTIN7P13)	0.50^2^	No matches found	Hepatocellular carcinoma [114] Urinary bladder cancer [115]
OR9I1	0.59^2^	No matches found	Human keratinocytes [116]
CNNM3-DT	0.60^2^	Disease: Rheumatoid arthritis Disease Class: Skin and Connective Tissue Diseases; Musculoskeletal Diseases; Immune System Diseases [117]	Lipid metabolism [118]
ENDOU	0.61^2^	Disease: Mental depression Disease Class: Behavior Mechanisms [119]	Skin diseases [120]
		Disease: Depressive disorder Disease Class: Mental Disorders [121]	
		Disease: Depressed mood Disease Class: Behavior and Behavior Mechanisms [122]	

^1^Upregulated DEGs

^2^Downregulated DEGs

## Discussion

Sunlight is a source of ultraviolet (UV) radiation, which can harm the skin. Skin aging, which results from exposure to UV radiation, is often classified into three major categories (i.e., UV-A, UV-B, and UV-C). UV-A makes up 95% of the UV rays that reach the Earth’s surface, and UV-B makes up the remaining 5% [30]. UV-C is absorbed by the ozone layer. Because of the small amount of UV-B that reaches the Earth, there is no substantial evidence, confirming that UV-B causes more damage than UV-A. UV-A and UV-B may both harm the DNA and proteins of cells [31, 32]. UV light causes reactive oxygen species (ROS) through oxidized lipids and proteins to be produced on the skin’s surface. These ROS initiate oxidative stress and mutations, thus accelerating skin aging and wrinkling. UV-A primarily promotes the production of ^1^O_2_, whereas both UV-A and UV-B cause the production of ^·^O_2_^−^ via the activation of NADPH oxidase [33–35]. Exposure to UV-B radiation induces erythema by upregulating the expression of cyclooxygenase-2 (COX-2), which stimulates the inflammation process [36].

As we age, we naturally produce less collagen and other skin components, resulting in reduced collagen synthesis and enhanced collagen breakdown. This causes the appearance of skin aging associated explicitly with dermal matrix alterations that may include wrinkling, stiffness, and weakness of skin aging, as well as loss of skin elasticity [37]. Through the release of interleukins (e.g., (IL)-1a and IL-6), the ^1^O_2_ produced by UV-A promotes matrix metalloproteinase (MMP-1) generation in human skin fibroblasts [38, 39] and decreases collagen synthesis [40].

The effects of several anti-oxidants on the skin and skin cells, including ascorbic acid, tocopherols, carotenoids, natural compounds, and polyphenols, are of great importance [41]. These chemicals are mainly responsible for ROS and DNA damage reduction generated on the skin’s surface. Among these, polyphenols are a kind of molecular structure generally extracted from plants with the structural feature of phenol units with anti-inflammatory and anti-oxidant properties. They are reported to have COX inhibition activity, promote resistance to oxidative stress, and stop skin aging [42]. Moreover, the anti-oxidant or anti-inflammatory activities of phenolic acids can be enhanced through the presence of methoxy (-OCH_3_), phenolic hydroxyl (-OH) groups, and carboxylic acid (–CH_2_COOH, –CH = CHCOOH, –COOH) [43].

To investigate the anti-oxidant activity of the compounds, IC_50_ values are quantitatively measured to indicate how much of a particular inhibitory chemical is used to inhibit a biological component, such as an enzyme or receptor, by fifty percent. The higher anti-oxidant activities are in direct relationship with smaller IC_50_ values [43]. As listed in Table 3, the structures of Zingerone and similar compounds obtained from the results represent FDA-approved/experimental drugs and natural compounds. These chemicals with phenolic hydroxyl or methoxy groups automatically inherit the anti-oxidant and anti-inflammatory activities that prevent skin aging. On the other hand, their IC_50_ values were extracted from bindingdb.org, a public database of measured binding affinities [44, 45]. The extracted pIC_50_ =  − LOG_10_(IC_50_) values for eight structurally similar compounds and Zingerone range from 3.8 to 4.5 in different target/enzyme environments, representing their high anti-oxidant and anti-inflammatory activities. The primary mechanism of action of these compounds in terms of their anti-oxidant features is the direct scavenging of free radicals. The radical oxidation of anti-oxidants results in a more stable, less radical reaction. By interacting with the reactive radicals, anti-oxidants stabilize the ROS.

**Table 3:** Structure activity relationships of similar compounds and their targets/enzymes

Compound	Structure	IC50 (nM) (BindingDB.org)	Target/enzyme from DrugBank and literature	
			Target	Enzyme
Nabumetone		> 5.00E + 4 Estrogen receptor	Prostaglandin G/H synthase 2 [123] [124] Prostaglandin G/H synthase 1 [125, 126]	Myeloperoxidase [127]
Masoprocol		> 5.00E + 4 Androgen receptor	Arachidonate 5-lipoxygenase [128] [129] Sex hormone-binding globulin [130]	Arachidonate 5-lipoxygenase [131]
Matairesinol		5.20E + 4 Testis-specific androgen-binding protein	Dehydrogenase/reductase SDR family member 4-like 2 [132]	N/A
3-(4-hydroxyphenyl)-1-(2,4,6-trihydroxyphenyl)propan-1-one (Phloretin)		1.67E + 5 Topoisomerase I/II	HTH-type transcriptional regulator TtgR [132]	N/A
6-Paradol		> 3.00E + 4 Cytochrome P450 3A	Pain receptor [133]	COX1 [133] Capsaicin [133]
6-Shogaol		9.96E + 4 Cytochrome P450 2E1	Prostaglandin E [134] NF-κB [135]	Phase II genes expression enzymes [136]
6-Gingerol		1.29E + 5 Cyclooxygenase-1 (COX-1)	Cell growth regulatory proteins [135] NF-κB [135]	COX-2 [137] Extracellular signal-regulated kinases (ERK) [135]
Zingerone		1.53E + 5 Androgen Receptor	Peroxisome proliferator-activated receptor alpha [138]	Xanthine oxidase [8] Acetyl-CoA carboxylase [138] Acetyl-CoA synthetase [138]

The methoxy and hydroxyl groups of the anti-oxidants with the highest reactivity can make free radicals inactive (i.e., Eq. (1)):

1 $${\text{Phenolic}}\;{\text{anti - oxidant}}\;\left( {{\text{OH}}\;{\text{or}}\;{\text{OCH}}_{{3}} \;{\text{or}}\; \ldots } \right) + {\text{R}}^{ \cdot } \to {\text{Phenolic}}\;{\text{anti - oxidant}}\;\left( {{\text{O}}^{ \cdot } \;{\text{or}}\;{\text{OCH}}_{{{2}^{ \cdot } }} \;{\text{or}}\; \ldots } \right) + {\text{RH}}$$

where R^·^ is a type of free radical such as hydroxyl, peroxyl, alkoxyl, or alkyl radicals and O^·^ or OCH_2_^·^ are the remained free radicals [46, 47]. Similarly, the mechanism of action of these chemicals, given their anti-inflammatory properties, applies through radical scavenging activities [48].

Additionally, it has been established that the immune system can function in the human body as a double-edged sword in which immunity and immunopathology simultaneously provide benefits and do damage by balancing innate and adaptive immunity [49]. In a recent review, Zouboulis et al*.* stated that “In clinical practice, ‘to look better’ does not mean to ‘look younger.’” [50].

Skin, the largest organ of the human body, inherits all immune system functions for better studying inflammation, autoimmunity, and cancer [51]. Skin aging is a multistep process that can be promoted through sun exposure, which may result in epidermal changes and photo-aging [52]. Various studies have proposed the benefits of natural compounds such as curcumin and its analogs in treating skin disorders such as skin aging [53, 54].

In the current investigation, the effects of Zingerone on epidermal tissues have been studied while considering the differentially expressed genes and the cellular components involved. As an outcome, without any exceptions, the assessed genes play roles in involving interleukins, which are a well-known group of cytokines expressed mainly by leukocytes. Cytokines, which are produced by the Langerhans cells of the skin’s immune system, were reported to play a significant role in skin aging [55]. Based on the identified differentially expressed genes and their effective cellular components in terms of GO, including GO:0005615 and GO:0005576, eighteen and four types of interleukins were determined to play roles in extracellular space and region, respectively.

In line with our results, Shirato et al*.* found that ETAS 50 could prevent skin aging by decreasing both UV-B-induced IL-6 and IL-1 beta expressions (56, 57). Also, Guo et al*.* reported that adipose-derived stem cells could secrete several interleukins (e.g., interleukin-lβ, interleukin-8, interleukin-9, interleukin-12, interleukin-15, and interleukin-17) to inhibit skin aging [58]. Exercising could also affect interleukin-15 levels, thus preventing skin aging [59]. Interleukin 17 (or IL-17A) has a direct relationship with the stimulation of IL-23, making them a golden IL-23/IL-17 axis in age-associated inflammation and attenuates skin aging via acetyl Zingerone treatment through IL-17A stimulation [60, 61].

In summary, the current study proposed a hybrid systems biology and systems pharmacology procedure to identify the functional mechanisms and successful effects of Zingerone treatment. The proposed procedure considers the impact of similar compounds for stimulating the involved interleukins family on skin aging and their anti-oxidant and anti-inflammatory properties. More experimental studies on human and animal models are required to confirm the present results, as Zingerone’s effects on cellular and molecular mechanisms on skin aging are still unclear.

## Conclusions

The prominent role of herbal remedies is clear to the research and scientific communities. Skin aging and other types of skin disorders have attracted the attention of people throughout the world. In this regard, several drugs and chemicals have been proposed for anti-aging purposes. In this study, a computational and statistical procedure was considered to investigate the effect of Zingerone on skin aging at the cellular and genomic levels. Additionally, compounds that are structurally similar to Zingerone and their impacts on skin-related diseases were studied.

Furthermore, the gene–disease association and gene–chemicals network analyses revealed undeniable direct connections between genetics and skin disorders, including skin aging. Finally, various types of interleukins were found to have vital roles in attenuating skin aging. However, further research using human or animal models is required to confirm the effects of Zingerone determined in the present study.

## Data Availability

Not applicable.

## Abbreviations

DEG: Differentially expressed genes
GEO: Gene expression omnibus
GO: Gene ontology
KEGG: Kyoto encyclopedia of genes and genomes
NCBI: National Center for Biotechnology Information
RHE: Reconstructed human epidermal
SMILES: Simplified Molecular Input Line Entry System
FDA: Food and Drug Administration (US)
ADME: Absorption, distribution, metabolism, and excretion
CTSL: Cathepsin L
RFPL4AL1: Ret Finger Protein Like 4A Like 1
TNFRSF11B: TNF Receptor Superfamily Member 11b
SPACA5: Sperm Acrosome Associated 5
TMEM158: Transmembrane Protein 158
DNLZ: DNL-Type Zinc Finger
MIR614: MicroRNA 614
MIR1282: MicroRNA 1282
MIR4469: MicroRNA 4469
LOC100287497 (SEPTIN7P13): Septin 7 Pseudogene 13
OR9I1: Olfactory Receptor Family 9 Subfamily I Member 1
CNNM3-DT: CNNM3 Divergent Transcript
ENDOU: Endonuclease, Poly(U) Specific
UV: Ultraviolet radiation
ROS: Reactive oxygen species
NADPH: Nicotinamide adenine dinucleotide phosphate
